# Navigating identity and professional life: A qualitative study of LGBTQ+ genetic counselors' workplace experiences

**DOI:** 10.1002/jgc4.70175

**Published:** 2026-01-30

**Authors:** Kayla L. Nelson, Kimberly Zayhowski, Ian M. MacFarlane

**Affiliations:** ^1^ Department of Genetics, Cell Biology, and Development University of Minnesota – Twin Cities Minneapolis Minnesota USA; ^2^ Medical Sciences and Education, Boston University Chobanian and Avedisian School of Medicine Boston Massachusetts USA

**Keywords:** genetic counselors, identity disclosure, LGBTQ+, minority tax, qualitative research, workplace climate

## Abstract

Lesbian, gay, bisexual, transgender, and queer/questioning (LGBTQ+) healthcare professionals frequently navigate complex dynamics related to their professional identities, including disclosure, advocacy, and experiences of minority stress. This qualitative study explored how LGBTQ+ genetic counselors manage their professional identities, including decisions about disclosure, advocacy and representation, and encounters with discrimination, within workplace and broader sociopolitical contexts. Twelve LGBTQ+ genetic counselors practicing in the United States participated in semi‐structured interviews about their workplace experiences and identity management processes. Using a constructivist framework and reflexive thematic analysis, we conceptualized three overarching themes: (1) Navigating disclosure—Environmental signals and professional outness; (2) The burden and opportunity of being “The LGBTQ+ Expert”; (3) Enduring microaggressions and workplace disillusionment. Participants described ongoing assessments of risk and benefit in disclosure decisions, balancing authenticity with professionalism, and navigating institutional and sociopolitical pressures. They reported that advocacy and visibility often brought both pride and additional labor, and that microaggressions and performative inclusivity contributed to chronic disillusionment. These findings underscored the importance of understanding disclosure dynamics and implementing strategies to promote inclusion, belonging, and equity for LGBTQ+ professionals in genetic counseling.

**What is known about this topic**

LGBTQ+ healthcare professionals navigate complex decisions around identity disclosure at work, balancing authenticity with perceived safety, professionalism, and potential risks. Disclosure can impact experiences of discrimination, minority stress, and professional burnout.

**What this paper adds to the topic**

This study provides an in‐depth exploration of LGBTQ+ genetic counselors' workplace experiences, highlighting the dynamic negotiation of disclosure, the dual burden and opportunity of being viewed as “The LGBTQ+ Expert,” and the enduring impact of microaggressions and workplace disillusionment.

## 1 INTRODUCTION

Between 2012 and 2023, the percentage of adults in the U.S. who openly identify as lesbian, gay, bisexual, transgender, and queer/questioning (LGBTQ+) grew from 3.5% to 7.6% (Jones, 2024). According to the 2024 Professional Status Survey (PSS) by the National Society of Genetic Counselors (NSGC), 86% of genetic counselor (GC) respondents identified as heterosexual/straight, while 6% identified as bisexual, 3% as gay or lesbian, 2% as queer, and 1% each as pansexual/omnisexual/polysexual, questioning, and asexual. Additionally, 4% of respondents identified outside the cisgender binary, bringing the total reported percentage of LGBTQ+ individuals within the genetic counseling profession above the national average. These characteristics make it important to examine LGBTQ+ experiences specifically within the genetic counseling profession, where clinicians' identities can directly shape counseling encounters and small, tightly networked career structures.

The majority of published LGBTQ+ genetic counseling research has focused on patient experiences, particularly those of transgender patients (Barnes et al., 2020; Bland et al., 2023; Huser et al., 2022; Rolle et al., 2022; Ruderman et al., 2021; Sacca et al., 2019; Schechner et al., 2025; Sutherland et al., 2020; von Vaupel‐Klein & Walsh, 2021; Zayhowski et al., 2019; Zayhowski, Horowitz, et al., 2025). To further promote well‐informed genetic counseling for LGBTQ+ patients, one study proposed LGBTQ+ competencies to be used in genetic counseling graduate programs' curricula (Saunders et al., 2025). Despite increased efforts to improve inclusivity for patients, LGBTQ+ genetic counselors have reported lower satisfaction with NSGC's inclusivity efforts compared to their heterosexual/straight peers. Specifically, a Diversity, Equity, and Inclusion Assessment for NSGC showed that 17% of LGBTQ+ GCs were dissatisfied or very dissatisfied with diversity and inclusion within the field, versus 8% of straight/heterosexual GCs (National Society of Genetic Counselors, 2022). Further, research on graduate program application experiences revealed LGBTQ+ GCs feared disclosing their identity due to concerns about discrimination and lack of diversity within the profession (O'Sullivan et al., 2023). Similarly, Chu et al. (2024) found LGBTQ+ GC students described how faculty and classmates reinforced cisheteronormative environments, with recruitment efforts emphasizing diversity over genuine inclusion.

Disclosing one's LGBTQ+ identity is an ongoing and lifelong process (Quinn, 2017). LGBTQ+ health professionals report that navigating between disclosure and concealment is a continuous and exhausting challenge that involves frequent weighing of risks and benefits (Beagan et al., 2022, 2023). Past research has highlighted students' hesitation to disclose their identities while applying to, and during, genetic counseling graduate programs, stemming from isolation, formal faculty‐peer relationships, and fear of discrimination (Chu et al., 2024; O'Sullivan et al., 2023). The increased risk of discrimination significantly impacts decisions to disclose. For instance, Casey et al. (2019) found that over half of LGBTQ+ adults have experienced discrimination through slurs, microaggressions, sexual harassment, or violence. Brady et al. (2022) identified a positive association between fear of heterosexism and discomfort with identity disclosure among LGBTQ+ university students; “heterosexism” refers to the psychosocial and emotional responses to potential bias encountered in familial or social settings. Additionally, concealment or disclosure decisions can negatively influence job satisfaction and feelings of belonging (Newheiser et al., 2017). While other fields have explored LGBTQ+ professionals' workplace experiences, no published research has specifically addressed LGBTQ+ GCs' workplace experiences to the authors' knowledge.

Research in other health professions demonstrates that LGBTQ+ identity disclosure may lead to workplace discrimination, threats to physical safety, burnout, and jeopardized job security (Bizzeth & Beagan, 2023; Casey et al., 2019; Dentato et al., 2014; Nama et al., 2017; Suppes et al., 2021). LGBTQ+ individuals, especially transgender people, face increased risk of violent crime and sexual assault compared to cisgender individuals (Flores et al., 2021). Disclosure can also affect advancement opportunities; Casey et al. (2019) found that 19% of white LGBTQ+ individuals and 28% of LGBTQ+ individuals from racial/ethnic minorities reported discrimination when seeking equal pay or promotions. Underrepresented minorities in medicine experience promotion disparities attributed to additional diversity‐related responsibilities, lack of mentorship, and greater isolation (Rodríguez et al., 2015). According to the 2024 Human Rights Campaign State Equality Index, 32 states have employment laws prohibiting discrimination based on sexual orientation and gender identity, and one state prohibits discrimination based on sexual orientation only (HRC Foundation, 2025).

The terms minority tax and cultural taxation refer to the extra responsibilities and burdens placed on minority professionals in the name of diversity (Rodríguez et al., 2015). Most minority tax research has focused on racial and ethnic identities among underrepresented minorities (Kamceva et al., 2022; Mahoney et al., 2008; Rodríguez et al., 2015), identifying themes of responsibility for diversity efforts, experiences of discrimination and isolation, and lack of promotion or mentorship. Although initial research centered on racial/ethnic identity, authors have suggested that minority tax dynamics apply to other underrepresented groups, including LGBTQ+ communities (Kamceva et al., 2022; Layne et al., 2022; Mahoney et al., 2008; Rodríguez et al., 2015; Sánchez et al., 2013).

Given the well‐documented experiences of LGBTQ+ professionals in other fields, it is important to assess whether LGBTQ+ genetic counselors report similar challenges. Understanding these experiences is essential for promoting a supportive, safe, and inclusive workplace for LGBTQ+ GCs, and can also improve care and access for LGBTQ+ patients. This qualitative study explores how LGBTQ+ genetic counselors navigate professional identity, including disclosure at work, advocacy, and experiences of discrimination within the genetic counseling profession.

## 2 METHODOLOGY

In this qualitative study, we conducted semi‐structured interviews to gather information about the experiences of LGBTQ+ GCs in the workplace. A constructivist framework and reflexive thematic analysis approach were used to analyze the data and generate themes via inductive coding. The University of Minnesota Institutional Review Board approved this study (STUDY00020303). Two members of the research team are LGBTQ+. Our research team has expertise in genetic counseling and genetic counseling training, LGBTQ+ research, and qualitative research methods. At the time the study was conducted, the first author was a genetic counseling graduate student. The first author received training on reflexive thematic analysis by the second and third author, who both have experience using reflexive thematic analysis in prior studies.

### 2.1 Participants

Eligible participants for this study were individuals who are LGBTQ+ and practicing GCs in the United States or Canada. Eligibility was self‐reported via an initial screening survey (see Appendix S1). We recruited participants primarily through posts on a dedicated Slack channel for LGBTQ+ genetic counselors. We purposively selected interviewees to maximize representation across LGBTQ+ identities. The interviewer did not have any prior relationship with the participants. We ceased recruitment when we determined sufficient informational power, meaning the sample provided adequate depth, diversity, and relevance to the study aim (Malterud et al., 2016).

### 2.2 Data generation

The initial screening survey collected demographic information (age, pronouns, race/ethnicity, gender identity, sexual orientation), workplace details (country and state, province, or territory of practice, type of work environment, years of genetic counseling practice, practice setting), and contact information.

Our team created a semi‐structured interview guide based on previous existing literature regarding the experiences of racial/ethnic minorities in medicine, LGBTQ+ undergraduate and graduate students including genetic counseling students, LGBTQ+ medical students, LGBTQ+ professors, and LGBTQ+ healthcare workers. The interview guide was piloted with an LGBTQ+ GC on the research team. Following the pilot, we edited several questions to improve clarity and to include inquiry about changes in disclosure decision‐making among participants who had experience in multiple jobs.

The final interview guide (see Appendix S2) included questions on the influence of LGBTQ+ identities on job applications and interviews, factors affecting disclosure of LGBTQ+ identity in the workplace, definitions of being “professionally out,” the role of LGBTQ+ identity in Diversity, Equity, Inclusion, and Justice (DEIJ) initiatives, workplace experiences of LGBTQ+ genetic counselors, and the impact of intersecting identities on these experiences.

Prospective participants completed an initial screening survey via Qualtrics to confirm eligibility, provide informed consent, and indicate their willingness to be contacted for a Zoom interview by supplying their email address. The first author scheduled interviews using email correspondence and conducted them one‐on‐one via Zoom, a secure video conferencing platform, from November to December 2023. Only the participant and interviewer were present during interviews, which were conducted in the first author's home office. With participant consent, interviews were audio‐recorded for transcription and analysis. Before recording began, the first author self‐disclosed her LGBTQ+ identity to promote trust and comfort with participants, acknowledging the relational context integral to reflexive thematic analysis. Throughout the interview and analysis process, the first author engaged in reflexive journaling to document thoughts, assumptions, and decisions. Interview duration ranged from 30 to 90 min (average: 48 min). Audio files were stored securely on the University of Minnesota's Box Secure Storage platform. Participants were mailed a $20 gift card as remuneration.

### 2.3 Data analysis

Audio recordings were transcribed verbatim by the first author, with all transcripts uploaded to the University of Minnesota's Box Secure Storage platform. Identifying information was redacted to protect confidentiality. To ensure transcription accuracy, the third author audited randomly selected 5‐min sections, comparing transcripts to audio recordings. Transcripts were imported into NVivo (version 14.23.2) for qualitative data analysis.

Adopting a constructivist perspective and reflexive thematic analysis approach, the first author engaged closely and iteratively with the data as each transcript was completed, generating initial codes and developing patterns of meaning (Braun & Clarke, 2006, 2021, 2024). This specific methodological approach was used to conceptualize themes for a more comprehensive understanding of LGBTQ+ genetic counselors' lived experiences within the workplace. Coding and theme development were recursive, involving movement between individual data extracts and the broader data set to ensure coherence and depth. We collated relevant data extracts with developing themes and constructed a thematic map to assist with the evolving analysis. As an author team, we collaboratively refined theme names and definitions as the analysis progressed. To further protect participant confidentiality, quotes included in the findings were not linked to participant numbers; the number of quotes per participant ranged from 1 to 4. Our author team discussed thematic maps and evolving interpretations throughout the analytic process, consistent with a reflexive and collaborative approach.

## 3 ANALYSIS

Twenty‐eight people completed the initial screening survey and consented to participate in this study. Twelve participants were selected to partake in one‐on‐one interviews. A majority of participants were non‐binary (*n* = 6), queer (*n* = 5), white (including those who were multiracial with white selected; *n* = 9), and 20–29 years of age (*n* = 7). All 12 participants were practicing within the United States. Further participant demographic information can be found in Table 1.

**TABLE 1:** Participant demographic information (*N* = 12).

Demographic		Participants (*n*)
Age	20–29 years	7
	30–39 years	3
	40–49 years	2
Sexual orientation^a^	Queer	5
	Pansexual	3
	Lesbian	2
	Gay	2
	Bisexual	2
Gender^a^	Non‐binary	6
	Cisgender woman	4
	Genderqueer	3
	Cisgender man	1
Race/ethnicity^a^	White	9
	Asian	3
	Hispanic, Latine, or of Spanish Origin	2
	Black or African American	1
	Prefer to self‐describe:	
	“Mixed race”	1
U.S. region of practice^b^	Pacific Region	6
	Northeast Region	3
	Southeast Region	2
	Southwest Region	1
Work environment	Hybrid	6
	In‐person	4
	Remote	2
Setting of practice^a^	Clinical	10
	Education	1
	Not specified	1
Years of practice	0–5 years	7
	6–10 years	1
	11–15 years	4

^a^ Participants were invited to provide multiple descriptors and/or write in self‐descriptions.

^b^ U.S. states were grouped by region to protect participant anonymity.

We conceptualized three major themes from the data: (1) Navigating disclosure—Environmental signals and professional outness; (2) The burden and opportunity of being “The LGBTQ+ Expert”; (3) Enduring microaggressions and workplace disillusionment.

### 3.1 Theme 1: Navigating disclosure—Environmental signals and professional outness

LGBTQ+ genetic counselors described disclosure as an ongoing, dynamic process, shaped by continual assessment of safety, authenticity, and external risks. Decisions about whether, when, and how to disclose their identities were influenced both by environmental cues, including workplace culture, institutional policies, and broader sociopolitical factors, and by personal negotiation of professional visibility, authenticity, and norms. Disclosure was rarely a single moment; instead, it was fluid, evolving across settings, relationships, and experiences.

#### 3.1.1 Subtheme 1: Reading and responding to environmental signals

Participants described ongoing vigilance in assessing workplace safety and comfort. This process included interpreting institutional cues, such as DEIJ statements and inclusive language policies, as well as evaluating direct social interactions with colleagues and supervisors.

Part of the application process for [one job] was to provide a DEIJ … statement as part of your application, and so that at least tells me that they're thinking about recruiting diverse individuals. But that doesn't necessarily always translate to a positive work environment. … In the job search you can't always get a perfect idea of the values of folks who work there, or what the actual workplace culture is like.

Multiple participants included the relative normativity of inclusive practices like introducing oneself with one's pronouns even if that individual is not part of the LGBTQ+ community.

One of the biggest things [I look for] when people are going around and introducing themselves. Are they introducing themselves with pronouns? Are they using my correct pronouns when they're referring to me? I go by a name that is not my legal name, and I asked in interviews if they thought that'd be a problem with their system, if they'd had to handle that before. … Those kinds of questions were easier for me to ask than things like, ‘How accepting of an environment are you?’

One participant described how a colleague initially communicated acceptance, but later on demonstrated the opposite.

[That experience] made it difficult in figuring out who is a safe space to talk to. People can say they're a safe space … and then you share information, and they are not a safe space. … I've just had some negative experiences. I've just been a lot more wary about how I talk about [my identities], and how I bring it up.

Peer and supervisor responses to inclusive language efforts or DEIJ workshops often signaled the limits of genuine inclusivity.

In [a DEIJ] workshop, I felt the need to disclose my identity because there were some providers who … [were] not overtly against the efforts but were like, 'Why do we need to put so much effort into DEIJ initiatives … and why does it need to be so politicized?' … I felt the need to step forward and say our identities have always been politicized and it's not us that is making this political. It's the systems at play that insist on politicizing our existence.

The reactions of others to diversity initiatives or inclusive care practices, especially from those in majority identities or positions of authority, held significant weight in disclosure decisions.

When we were first piloting this inclusive language initiative, the pushback I was getting was from my colleagues who were older, … white males, and they were the ones saying things like, ‘Well, you're reducing what a woman really is by using things like person who is pregnant.’ … But there was almost … an accusation of removing womanhood when we're just trying to include personhood.

The external sociopolitical context, including state and federal legislation, also powerfully shaped counselors' perceptions of safety and affected both disclosure and career decisions. Many described how anti‐LGBTQ+ laws affected their mental health, sense of belonging, and practical life choices.

I'm now in the deep South, and that … has changed my experience a little bit on my perception about location for job. … There's been so much anti‐trans laws. There's been like 500 in the past three years. It's insane. And just with all of that, location is a huge piece.

Every legislative season … is pretty stressful because there is usually 20 or more anti‐LGBTQ bills introduced into the state legislature. Only a handful have really passed. … It does make it scary to live here. … And if Obergefell v. Hodges were overturned, which has been threatened, especially since the Dobbs case, … my wife and I are expecting our marriage license to be revoked.

#### 3.1.2 Subtheme 2: Negotiating professional outness and identity management

Participants described an ongoing negotiation of visibility, authenticity, and professionalism. Professional outness was a fluid, context‐dependent process, requiring careful calibration to balance authenticity with institutional expectations of “professionalism” rooted in cisheteronormative norms. This balancing act demanded emotional labor, as participants weighed the desire to be genuine against concerns about safety, timing, and potential bias. Participants described a range of perspectives on what it means to be professionally out. For some, it involved explicit disclosure, such as introducing pronouns or mentioning a same‐gender partner; for others, it was more implicit, reflecting the belief that their identity should not require acknowledgment.

I wish you just didn't have [to come out]. … It would be lovely to just not have to think twice about if a straight colleague mentions, ‘Oh yeah, I went to such and such with my boyfriend this weekend.’ [They] don't even think twice about it.

Not one of my straight colleagues, …has come out as straight. If people want to come out, I have no problem with folks knowing about me. … But I also have to stop and ask myself, ‘Why do people have to?’

Others reflected on how the concept of coming out had evolved over time.

When I was younger … it had to be some big announcement. Now I think of coming out as if I join a Zoom call and my pronouns are in my Zoom, then was that coming out? Does that count as that?

Managing visibility required balancing authenticity, timing, and norms. Participants described challenges in finding natural ways to disclose, especially when straight‐passing in relationships.

There's not just a natural way for me to just announce it. … There's something different about it being a professional work setting. … That's why I feel I was just waiting for it to more organically come up so that it doesn't feel like a big thing.

It was not an uncommon experience for participants to describe feeling the need to share their identity in a deliberate manner in order to appease others. One participant described changing their pronouns but choosing not to correct their colleagues because colleagues explicitly told them that doing so could make others “uncomfortable.” They noted that disclosure risks misgendering and questioned whether correcting colleagues was worth the potential disruption and emotional burden.

We're told and expected to be very careful [about disclosure]. We don't want to make anybody uncomfortable. We don't want to draw excessive attention.

This same participant went on to share that they held a different approach with the students they work with.

I knew that when I changed my pronouns, some of the students were gonna come and ask me questions, and I welcomed that. I didn't want to make a big thing about it. … I wanted to do it … in a way that let them know that this is who I am, and if they wanted to talk about it, that I was open to do it. … You should be open to some level of discourse with the students that I don't feel my colleagues are entitled to.

Some participants worried that sharing their identity might be seen as unprofessional or distracting.

Professionalism carries such a connotation to it of underlying racism, misogyny, queerphobia, and everything that being open about one's sexuality, … people automatically think about sexual encounters. Their mind automatically goes to that.

I've been struggling … on the topic of professionalism in our profession. … I always second guess, ‘Was that professional?’ When you have someone that tells you [coming out to them] made a huge impact, then I'm like I don't care if someone else thinks it's professional or not because this is more important, right?

Still, several participants resisted those norms and emphasized that being open about their identities was essential to authenticity, connection, and inclusive care.

Telling the person that you should hide your identity to protect yourself is also an aggression and not okay. And yet it's actually really common in GC training and supervised relationships of, ‘You should look more professional. You should wear X. You shouldn't have your natural hair. You shouldn't have tattoos. You should hide all the unique aspects of yourself to be professional [and] to make sure patients don't react badly to you.’ That's all coming from places of every single phobia out there.

### 3.2 Theme 2: The burden and opportunity of being “The LGBTQ+ Expert”

Across interviews, participants described how being openly LGBTQ+ in the workplace often positioned them, implicitly or explicitly, as educators, advocates, and representatives for the broader LGBTQ+ community. This positioning brought both pride and purpose, offering opportunities to model inclusion, mentor others, and advance affirming care practices.

I'm definitely a big proponent of existence is resistance. Sometimes I give guest lectures on how to be an advocate and find your ways into advocacy spaces, and especially for marginalized people. Sometimes it doesn't have to be as obvious as being a volunteer on a SIG [special interest group] or being part of a committee, and sometimes it's just the things you're doing every day like making your own clinic materials more inclusive. That is a form of advocacy.

At the same time, the responsibility to educate and represent introduced strain and frustration. Participants often felt pressure to initiate or sustain diversity and inclusion efforts that were not equally shared by cisgender and straight colleagues. Many described this as a form of “minority tax,” in which their visibility and expertise became additional, often unacknowledged, emotional and professional labor.

People are just like, ‘Oh, I want to learn more about X. Tell me.’ And I'm like learn what? For what purpose? What have you Googled so far or tried to learn on your own? In those cases that feels like pressure.

My belief is also, if I'm not gonna do it, who is? … I unfortunately can't really trust other people to have that initiative to speak up if they're not part of those communities.

Participants were broadly asked about disclosure in the workplace, and several participants discussed disclosure to patients and students. Importantly, participants described how their LGBTQ+ identity could enhance their professional skills and relationships. Being out and visible allowed them to promote trust, empathy, and safety with patients and students, particularly those with marginalized identities. Disclosure, when thoughtfully managed, became a tool for patient‐centered care, signaling inclusivity and understanding.

When it comes to disclosing to a patient, the potential benefit is the patient realizing that I am a safe space to hold their minoritized identities. Maybe a patient will fear less of being discriminated against because of what their partnership looks like, or what their identity looks like, or what their gender identity has changed to be. Maybe they will feel safer in my presence.

I'm very grateful that … I'm able to provide representation, … or that somebody can already feel potentially comfortable with [the LGBTQ+ genetic counselor] going into something like a genetic counseling session. … I'm having that information [my LGBTQ+ identity] on the [place of work's] website so that [patients] feel like they can talk to somebody who understands.

Participants reported similar benefits with students.

I almost universally across the board do disclose my identity to my students … on purpose. … I want them to know … that there is somebody else who maybe understands a little bit of what they're going through. … I've found after [I self‐disclose], a lot of students will come up and disclose to me. … I have only seen … a net positive benefit to intentionally disclosing to my students.

Participants highlighted that their lived experiences informed psychosocial skills and cultural awareness that might not come as intuitively to cisgender or straight colleagues.

My queer identity helps inform my sense of empathy in how I approach conversations with patients. … Particular nuances that come with being able to show proper respect and how to have proper interactions with trans people in the clinic space that comes from having a large friend group of queer people and working with many queer people in the past. That may not be as intuitive, or come as easy, to cisgender or heterosexual colleagues.

Finally, holding an LGBTQ+ identity enhanced attention to language and inclusivity within the genetic counseling session.

I'm able to think about the language we use in different ways because I'm always thinking about how we can affirm people's gender when talking about genetics, which can be really binary. That's given me a cool skill set about being very intentional with the words I'm using with my patients and also tailoring a genetic counseling session.

Together, these findings illustrate the dual nature of being “The LGBTQ+ Expert”: opportunities for advocacy, mentorship, and enhanced patient care co‐exist with persistent burdens that counselors continually weigh in deciding when and how to engage.

### 3.3 Theme 3: Enduring microaggressions and workplace disillusionment

Despite efforts to promote inclusion, participants frequently encountered environments where subtle and overt forms of bias persisted. They described the cumulative toll of microaggressions, misgendering, performative allyship, and institutional inaction, which over time contributed to exhaustion, isolation, and disillusionment. For many, these experiences undermined their initial expectations of safety and belonging within ostensibly inclusive workplaces. Participants spoke to the emotional wear of navigating spaces that outwardly endorsed diversity yet failed to address the everyday realities of LGBTQ+ professionals.

I'm in a space where there aren't other people like that [LGBTQ+ allies], and it's very clear that either no one feels comfortable or confident enough to step up and say something, or other people just simply didn't realize it [the microaggression used] was wrong. … I'm already made more uncomfortable, and I can't or I might not want to speak up about certain things because now it seems like they won't be received [well].

Several non‐binary participants described various experiences of microaggressions in a workplace environment. Incorrect use of one's name or pronouns, for example, was a common experience for non‐binary GCs.

I don't think that anyone has maliciously misgendered me or continuously does. It's usually one moment, but I know that they've used the correct pronouns previously. … I'm used to that experience. I just choose to not open the can of worms. … I wish I were the kind of person who would say they/them every time that someone got it wrong. … It's so exhausting when people respond to it poorly that I just don't really want to get into it.

Participants described a sense of disillusionment stemming from inconsistencies between their organizations' stated commitment to DEIJ and their everyday experiences. One participant reported being told by a colleague that their response to a DEIJ initiative was them being “sensitive to the issue.” Another participant described the disconnect between their boss's public support for DEIJ and his actual behavior.

It's always more established or senior people that misgender me a lot at work. … [My boss] mislabeled me in a … meeting. … [The geneticist] had to correct him. … [My boss] was the person that initially was like, ‘We have great DEI initiatives and we really value diverse perspectives.’

Others echoed similar feelings, describing that their institutions were only superficially safe and inclusive, but fraught with microaggressions and hierarchical power dynamics.

You guys talk the talk, so I thought I was walking into a safe space, and then [it's] just a space filled with microaggressions and politics of hospitals and all those power dynamics. … I thought this was an accepting place, and it isn't. … And now I'm feeling very disillusioned and not feeling safe.

These accounts underscore how the persistent gap between institutional rhetoric and lived reality leaves individuals feeling unsafe, unsupported, and disillusioned.

## 4 DISCUSSION

This qualitative study provides in‐depth insight into how LGBTQ+ genetic counselors negotiate disclosure, a complex, ongoing process shaped by safety, authenticity, and workplace cues. Participants revealed concrete strategies for assessing inclusivity and illuminated persistent barriers such as cisheteronormativity and discrimination. These lived experiences highlight areas for reform to promote supportive professional environments.

### 4.1 Experiences of minority tax

Our findings reinforce that minority tax is a central challenge for LGBTQ+ genetic counselors, permeating all aspects of professional life. Disclosure is not a one‐time event; rather, it requires continuous, context‐dependent negotiation and risk assessment, shaped by the perceived safety of colleagues, patients, and students (Beagan et al., 2022, 2023). Participants described ongoing vigilance in assessing workplace cues, interpreting institutional signals such as DEIJ statements, inclusive language policies, and peer responses, as well as weighing subtle and overt social interactions. These assessments influence how, when, and to whom they disclose their LGBTQ+ identity, reflecting the dynamic and strategic nature of professional outness.

Participants also described the expectation to provide education on LGBTQ+ issues and representation—an educator role that disproportionately falls on minoritized individuals and mirrors patterns seen among BIPOC and disabled professionals (Carmichael et al., 2020; Chu et al., 2024; Epstein et al., 2023; Kamceva et al., 2022; Mahoney et al., 2008; Rodríguez et al., 2015; Schoonveld et al., 2007). Minority tax operates not only as an additional workload but as a subtle reinforcement of systemic inequities: visibility and perceived expertise may be rewarded socially but rarely translate into formal recognition, protected time, or compensation. Crucially, many participants articulated a paradox, feeling obliged to educate while simultaneously mistrusting colleagues outside the community to represent issues appropriately. This duality—pride and purpose versus strain and exhaustion—underscores the emotional and professional labor inherent in sustaining LGBTQ+ advocacy within largely cisheteronormative environments. We suggest that LGBTQ+ counselors not be assumed the default educators; they should be able to choose when to lead based on capacity and relevance. Ideally, allies can take responsibility for basic education and advocacy, with LGBTQ+ community members consulted or co‐leading when lived experience is essential.

Microaggressions were a routine experience, most notably misgendering, alongside instances of discrimination and dismissal by colleagues as “sensitive.” These acts contributed to emotional exhaustion and feelings of isolation, findings echoed in broader research on professional minority stress and burnout (Aamodt et al., 2021; Carmichael et al., 2020; Casey et al., 2019; Darr et al., 2023; Dyrbye et al., 2022; Epstein et al., 2023; Lawrence et al., 2022; Ramsey et al., 2024; Schoonveld et al., 2007). Despite these challenges, many participants noted that their LGBTQ+ identity promoted empathy, pride, and a strengthened patient‐centric approach to care, affirming previous research on the strengths and insights brought by minoritized professionals (Darr et al., 2023; Epstein et al., 2023; Schoonveld et al., 2007). Moreover, research indicates that genetic counseling students and recent graduates report higher support and satisfaction supervised by individuals who share similar identity characteristics (Peplow et al., 2024). Our findings suggest that minority tax is intertwined with the unique contributions that LGBTQ+ counselors bring to the profession, highlighting both structural inequities and opportunities for professional impact.

### 4.2 “Professionalism” and cisheteronormativity in the workplace

The concept of “professionalism” emerged as a powerful tool for marginalization within the largely homogeneous field of genetic counseling (Berro & Zayhowski, 2023; National Society of Genetic Counselors, 2024). Cisheteronormative norms have been present within genetic counseling since the establishment of the profession (Berro & Zayhowski, 2023). Participants indicated that disclosure of LGBTQ+ identities is uniquely scrutinized and often deemed unprofessional, whereas cisgender and straight colleagues routinely share personal details without consequence. This double standard, rooted in historically cisheteronormative norms, also impacts applicants and students' willingness to disclose their identities (Chu et al., 2024; O'Sullivan et al., 2023). The expectation to come out in an “organic” way and fear of discomfort or negative impact on rapport further restricts authentic self‐expression at work.

The findings from this study are consistent with past research discussing how LGBTQ+ identity disclosure is viewed as unprofessional by cisgender and straight colleagues because they perceive these identities as linked solely to sexual encounters (Bizzeth & Beagan, 2023). Participants described a continual negotiation of visibility and authenticity, constantly balancing the desire to be genuine with institutional and social expectations. LGBTQ+ identity disclosure was often monitored through the lens of perceived professionalism, with fears that colleagues, students, or patients might associate sexual orientation or gender identity with inappropriate behavior. Several participants resisted these norms, emphasizing that authentic disclosure was essential to forming connections, affirming patients and students, and modeling inclusive practice. Together, these findings illustrate that disclosure and professional outness are not simply individual decisions but are embedded in structural, cultural, and sociopolitical contexts, requiring strategic navigation and emotional labor.

### 4.3 Practice implications

The findings from this study underscore that promoting thriving, inclusive environments for LGBTQ+ genetic counselors requires more than performative gestures or policy statements; it requires structural, interpersonal, and cultural interventions that address the systemic forces shaping professional outness and minority tax. Creating spaces where LGBTQ+ counselors feel safe to disclose their identities involves consistent, everyday practices: routinely sharing pronouns, using correct names, modeling affirming behaviors, and visibly signaling allyship. Mistakes, such as misgendering, must be addressed swiftly and accountably to reinforce trust and belonging (Cooper et al., 2020; Sevelius et al., 2020).

Beyond these interpersonal strategies, institutions must recognize that the labor of representation, advocacy, and DEIJ initiatives should not be borne disproportionately by LGBTQ+ counselors. To reduce minority tax without compromising accuracy or safety, institutions should support accountable allyship and community involvement, for example through structured training, developing LGBTQ+ competencies, co‐led educational sessions, and formal role assignments with protected time, recognition, and/or compensation (Rodríguez et al., 2015; Saunders et al., 2025). Tracking outcomes such as training uptake and satisfaction among LGBTQ+ staff can help ensure delegation is effective and trusted. Equitable distribution of responsibilities, through inclusive clinic policies, active ally engagement, updated forms, and committee assignments, combined with formal recognition or compensation for additional diversity work, is essential to mitigate minority tax (Faucett et al., 2022; Garcia & Lopez, 2022; Saunders et al., 2025; Williamson et al., 2021; Yu et al., 2023). Mentorship and peer support structures further reinforce a culture of inclusion, providing guidance and emotional resources that buffer against isolation and burnout (Bizzeth & Beagan, 2023; Brunsma et al., 2017; Carmichael et al., 2021; Rodríguez et al., 2015; St John & Goulet, 2022; Sue et al., 2019). Importantly, such approaches acknowledge that inclusion is both an ethical imperative and a strategic necessity for sustaining a diverse and resilient genetic counseling workforce.

### 4.4 Study limitations

Several limitations should be considered when interpreting the results of this study. First, self‐selection bias may have influenced the participant pool; LGBTQ+ genetic counselors who were particularly motivated to share their workplace experiences, whether positive or negative, may have been more likely to participate, potentially limiting the representativeness of the sample. The study's findings do not elucidate the specific impact of holding multiple minoritized identities on LGBTQ+ GCs' experiences. Geographical factors and state‐level legislation may also have affected participation. Eligible genetic counselors practicing in states without statutory protections for sexual orientation and gender identity may have hesitated to participate due to concerns about confidentiality, potential workplace repercussions, or fear of being involuntarily outed. These potential barriers to participation are exemplified by this study's overall overrepresentation of participants that practice within the Northeast or Pacific regions of the U.S. Recruitment was limited to the LGBTQI+ Slack page, meaning participation was restricted to individuals with internet access, awareness of the Slack group, and access to online professional networks. This may have excluded those who are less connected to LGBTQ+ professional community spaces or who are not active on social media. Additionally, most participants were relatively young and early in their careers, which may influence comfort with disclosure and advocacy; age and career stage likely affect risk tolerance, perceived consequences, and willingness to speak publicly about one's identity. Lastly, while the study included non‐binary participants, there were no participants who self‐identified as intersex or as transgender men or women. The absence of these perspectives should be considered when interpreting the scope of the study's findings.

### 4.5 Future directions

Given that this is one of the first studies to explore the experiences of LGBTQ+ genetic counselors, there remains a need for additional research in this area. Because this study did not include participants who identified as intersex or as transgender men or women, future studies should specifically seek to include these groups, as their experiences may differ substantially from those of cisgender or non‐binary individuals. Further research could also focus on LGBTQ+ genetic counselors who are closeted or have not disclosed their identities in any professional context, addressing gaps left by this study's recruitment approach. We did not explicitly ask participants about disclosure to students in clinical rotations or patients, so future research should examine how LGBTQ+ genetic counselors navigate disclosure in supervisory roles and clinical encounters. Additionally, future work should examine the intersection of LGBTQ+ identity with other minoritized characteristics, such as race, ethnicity, or disability, to better understand how these intersecting identities shape workplace experiences.

It should be noted that we conducted this research prior to the recent surge of anti‐LGBTQ+ legislation and public discourse, which has escalated significantly in the United States since the time the study's data were generated (e.g. state‐level bans on gender‐affirming care for minors, restrictions on transgender participation in sports, and laws limiting discussion of sexual orientation and gender identity in schools) and has been documented in recent reviews of policy activity (HRC Foundation, 2025; Zayhowski, Roth, et al., 2025). The social and political climate at the time of data generation may have influenced participant experiences and willingness to disclose certain perspectives. As workplaces continue to implement or remove various DEIJ initiatives in response to these shifting policies, research that seeks to understand disclosure and workplace climate over time, especially amid changing sociopolitical contexts, will be valuable for understanding how professional environments and disclosure experiences evolve.

## 5 CONCLUSIONS

This study reveals that identity disclosure for LGBTQ+ genetic counselors is an ongoing negotiation, shaping every professional and personal interaction, from colleagues to patients to students. Decisions about whether, when, and how to disclose one's identity are dynamic and context‐dependent, influenced by workplace culture, institutional norms, and broader sociopolitical pressures. Notions of “professionalism” were disproportionately called into question when disclosure involved LGBTQ+ identities, reflecting cisheteronormative bias in workplace expectations. Participants frequently experienced minority tax through misgendering, discrimination, and the added responsibility of educating and representing LGBTQ+ issues. These intersecting challenges highlight the emotional labor required to navigate professional authenticity while maintaining safety and belonging. There remains an urgent need for systemic change: workplaces must move beyond performative inclusion to actively reduce minority tax, distribute advocacy responsibilities equitably, and cultivate environments where LGBTQ+ counselors can thrive authentically.

## AUTHOR CONTRIBUTIONS

**Kayla L. Nelson:** Conceptualization; data curation; formal analysis; investigation; methodology; project administration; resources; visualization; writing – original draft; writing – review and editing. **Kimberly Zayhowski:** Conceptualization; methodology; formal analysis; supervision; visualization; writing – original draft; writing – review and editing. **Ian M. MacFarlane:** Conceptualization; data curation; methodology; project administration; supervision; visualization; writing – review and editing.

## FUNDING INFORMATION

Funding for this project was provided by the University of Minnesota Genetic Counseling Program.

## CONFLICT OF INTEREST STATEMENT

Kayla L. Nelson and Ian M. MacFarlane declare that they have no conflicts of interest. Kimberly Zayhowski is a Deputy Editor and Director of Diversity, Equity, and Inclusion Integration for the Journal of Genetic Counseling and co‐author of this article. She was excluded from editorial decision‐making related to the acceptance of this article for publication in the journal.

## ETHICS STATEMENT

This research study was reviewed by the University of Minnesota Institutional Review Board and was granted approval as an exempt study in October 2023 (STUDY00020303). All procedures followed were in accordance with the ethical standards of the responsible committee on human experimentation (institutional and national) and with the Helsinki Declaration of 1975, as revised in 2000.

Animal studies: No non‐human animal studies were carried out by the authors for this article.

## PATIENT CONSENT STATEMENT

Verbal informed consent was obtained from all participants prior to their inclusion in this study.

## Supporting information


Appendix S1.

Appendix S2.

## Data Availability

Research data are not shared due to the remote risk of re‐identifying a participant.
