# Supplementary material for: Navigating identity and professional life: A qualitative study of LGBTQ+ genetic counselors' workplace experiences
Source: J Genet Couns. 2026 Jan 30;35(1):e70175. doi: 10.1002/jgc4.70175 (PMC12859179; doi:10.1002/jgc4.70175)
Supplement: Supplementary file 1 — Appendix S1. [file JGC4-35-0-s001.docx]

**Supplemental Material 1**

**Initial Screening Survey**

What is your current age?

- 18-29 years
- 30-39 years
- 40-49 years
- 50-59 years
- 60+ years

What are your gender pronouns?

- She/her
- He/him
- They/them
- She/they
- He/they
- Other (please describe)
- Prefer not to say

What race/ethnicity do you identify with? Select all that apply.

- American Indian or Alaskan Native
- Asian
- Black or African American
- Native Hawaiian or Other Pacific Islander
- White
- Some other race, ethnicity, or origin
- Prefer to self-describe
- Prefer not to say

Are you of Hispanic, Latino/a/x, or of Spanish origin? Select all that apply.

- No, not of Hispanic, Latino/a/x, or Spanish origin
- Yes, Mexican, Mexican American, Chicano/a/x
- Yes, Puerto Rican
- Yes, Cuban
- Yes, another Hispanic, Latino/a/x, or Spanish origin
- Some other race, ethnicity, or origin
- Prefer to self-describe
- Prefer not to say

What gender identity do you identify with? Select all that apply.

- Cisgender man
- Cisgender woman
- Transgender man
- Transgender woman
- Intersex
- Non-binary
- Genderqueer
- Agender
- Two Spirit
- Other (please describe)
- Prefer not to say

What sexual orientation identity do you identify with? Select all that apply.

- Lesbian
- Gay
- Bisexual
- Asexual
- Heterosexual/straight
- Pansexual
- Queer
- Questioning
- Other (please describe)
- Prefer not to say

What country do you primarily practice genetic counseling?

- United States
- Canada

What state do you primarily practice genetic counseling?

- Alabama
- Alaska
- Arizona
- Arkansas
- California
- Colorado
- Connecticut
- Delaware
- Florida
- Georgia
- Hawaii
- Idaho
- Illinois
- Indiana
- Iowa
- Kansas
- Kentucky
- Louisiana
- Maine
- Maryland
- Massachusetts
- Michigan
- Minnesota
- Mississippi
- Missouri
- Montana
- Nebraska
- Nevada
- New Hampshire
- New Jersey
- New Mexico
- New York
- North Carolina
- North Dakota
- Ohio
- Oklahoma
- Oregon
- Pennsylvania
- Rhode Island
- South Carolina
- South Dakota
- Tennessee
- Texas
- Utah
- Vermont
- Virginia
- Washington
- West Virginia
- Wisconsin
- Wyoming

What Canadian province or territory do you primarily practice genetic counseling?

- Alberta
- British Columbia
- Manitoba
- New Brunswick
- Newfoundland and Labrador
- Northwest Territories
- Nova Scotia
- Nunavut
- Ontario
- Prince Edward Island
- Quebec
- Saskatchewan
- Yukon

What type of work environment do you primarily practice genetic counseling in?

- In-person
- Remote
- Hybrid

How long, in total, have you been practicing as a genetic counselor?

- 0-5 years
- 6-10 years
- 11-15 years
- 16-20 years
- 20+ years

In what type of setting do you primarily practice genetic counseling?

- Clinical
- Laboratory
- Industry
- Research
- Other (please describe)

May we contact you to participate in an interview about your experiences?

- Yes
- No

Please type your email address below to provide contact information to the researchers for participation in this research study.
