# Supplementary material for: Navigating identity and professional life: A qualitative study of LGBTQ+ genetic counselors' workplace experiences
Source: J Genet Couns. 2026 Jan 30;35(1):e70175. doi: 10.1002/jgc4.70175 (PMC12859179; doi:10.1002/jgc4.70175)
Supplement: Supplementary file 2 — Appendix S2. [file JGC4-35-0-s002.docx]

**Supplemental Material 2**

**Semi-Structured Interview Guide**

1. How, if at all, did your LGBTQ+ identity influence what job(s) you have applied to?
   1. What were the major factors you considered when applying for a job?
   2. If you’ve applied to and have had more than one job, how has this changed?
2. Did you choose to disclose your LGBTQ+ identity in any job applications or interviews?
   1. Why did you choose to or choose not to disclose your LGBTQ+ identity?
   2. How did you determine who, if anyone, to disclose your identity to?
   3. Were there any individuals who influenced your decision to disclose or not to disclose your LGBTQ+ identity and if so, why?
3. What factors influence your decision-making to disclose or not disclose your LGBTQ+ identity while at work?
   1. What factor(s) determine who, if anyone, you disclose to?
      1. If you choose not to disclose at work, do you modify the way you act, speak, dress, etc. to avoid accidental or unintentional disclosure?
   2. What would you say are the benefits and risks of each?
   3. What type of emotion(s) arise when making these decisions?
4. Coming out to others is a continuous process, and this process is also very individualized. When thinking about working within the genetic counseling field, what do you consider to be “professionally” out?
   1. Could ask about genetic counselors, MDs, geneticists, nurses, nurse practitioners, or any other healthcare workers one interacts with at work
   2. (e.g. out to your coworker(s), out to your supervisor or boss, out in a professional organization like NSGC, out on social media, out while counseling a patient, etc.)
5. How, if at all, has your LGBTQ+ identity impacted your involvement in Diversity, Equity, Inclusion, and Belonging (DEIB) efforts within the field?
   1. What factors influence what you choose to or choose not to get involved in?
      1. How has this decision-making process impacted, if at all, your personal well-being?
   2. Have you ever felt a pressure to provide representation for the LGBTQ+ community within this field?
      1. If yes, in what ways?
6. Is there anything you felt you have had to do, or were expected to do, outside of your job duties because of your LGBTQ+ identity?
7. Have you ever felt you had to prove yourself or your abilities because of your LGBTQ+ identity?
8. When thinking about practicing as a genetic counselor, have you ever experienced burnout?
   1. If yes, what factor(s) do you think led to its development?
9. How, if at all, do you feel that your experience is different identifying as an LGBTQ+ genetic counselor compared to that of your cis and/or straight colleagues?
10. How, if at all, do you feel like your LGBTQ+ identity impacts your sense of being a genetic counselor?
11. Are there any other intersecting identities that impact your genetic counseling work environment experience?
    1. If yes, please expand.
12. Is there anything else about your experiences you would like to share?
